# Clinicopathological Features of Advanced Gastric Cancers which Were Misjudged and Subjected to Endoscopic Submucosal Dissection

**DOI:** 10.1155/2020/6525098

**Published:** 2020-03-02

**Authors:** Yorinari Ochiai, Daisuke Kikuchi, Naoko Inoshita, Junnosuke Hayasaka, Yugo Suzuki, Masami Tanaka, Kosuke Nomura, Hiroyuki Odagiri, Satoshi Yamashita, Akira Matsui, Toshiro Iizuka, Masanobu Kitagawa, Shu Hoteya

**Affiliations:** ^1^Department of Gastroenterology, Toranomon Hospital, 105-8470 Tokyo, Japan; ^2^Department of Pathology, Tokyo Medical and Dental University, 113-8519 Tokyo, Japan; ^3^Department of Pathology, Toranomon Hospital, 105-8470 Tokyo, Japan; ^4^Department of Pathology, Tokyo Metropolitan Geriatric Hospital, 173-0015 Tokyo, Japan

## Abstract

**Background and Aims:**

Endoscopic submucosal dissection (ESD) is widely performed for early gastric cancer (EGC). We have sometimes encountered gastric cancer lesions for which ESD was performed and at which pathologically advanced cancer was found. In this study, we performed clinicopathological examination of lesions whose endoscopic diagnosis and pathology differed substantially.

**Methods:**

ESD was performed for 2,194 gastric cancer lesions (1,753 cases) in our institute from April 2005 through March 2015. The vertical margin was positive or status unknown in 51 lesions (2.3%); among these, muscularis propria (MP) or deeper infiltration was identified in 6 lesions from specimens obtained during subsequent surgery. In 1 lesion with MP invasion, the vertical margin was negative. We evaluated the clinicopathological features of these 7 lesions and retrospectively reviewed endoscopic indicators of submucosal invasion for EGC on white light imaging (WLI), narrow-band imaging magnifying endoscopy (NBI-ME), and endoscopic ultrasonography (EUS) performed previously.

**Results:**

Average age was 73.2 ± 7.2 years, and all cases were men. The 7 lesions diagnosed as advanced cancer were 0.32% of 2,194 lesions and were all located in the U region (fundus). On retrospective review of endoscopic findings, 2 of 7 lesions on WBI, 3 of 6 lesions on NBI-ME, and 2 of 5 lesions on EUS met the criteria for indicating submucosal invasion of EGC. No lesions had findings on all 3 modalities.

**Conclusion:**

In rare cases, advanced gastric cancer could not be accurately diagnosed by endoscopy using various modalities. Each case had special characteristics making identification of deep infiltration difficult.

## 1. Introduction

Endoscopic submucosal dissection (ESD) is less-invasive compared with conventional surgical resection. In recent years, an ESD procedure has been established and is widely used to treat early gastric cancer. Gotoda et al. stratified the risk of lymph node metastasis on the basis of pathological results from surgical resection specimens [1]. Their results identified lesions with a very low risk of metastasis, which are now considered to be indications for endoscopic treatment [2]. Findings from prospective studies have expanded the types of lesions that are indicated for endoscopic treatment [3, 4]. Although various factors have been reported to correlate with lymph node metastasis in gastric cancer, depth of invasion is one of the most important factors. Approaches used to infer tumor depth include white light (conventional) endoscopy, endoscopic ultrasonography (EUS), and narrow-band imaging magnifying endoscopy (NBI-ME); however, cases are sometimes misdiagnosed, usually as either mucosal (M) or submucosal (SM) cancer. In rare cases, lesions diagnosed as early cancer are revealed to be pathologically advanced after ESD is performed. In this study, we conducted clinicopathological examinations of cases in which pathological infiltration of the muscularis propria (MP) layer resulted in a diagnosis of advanced cancer despite being diagnosed as early gastric cancer before ESD.

## 2. Materials and Methods

### 2.1. Patients

**Figure 1:**
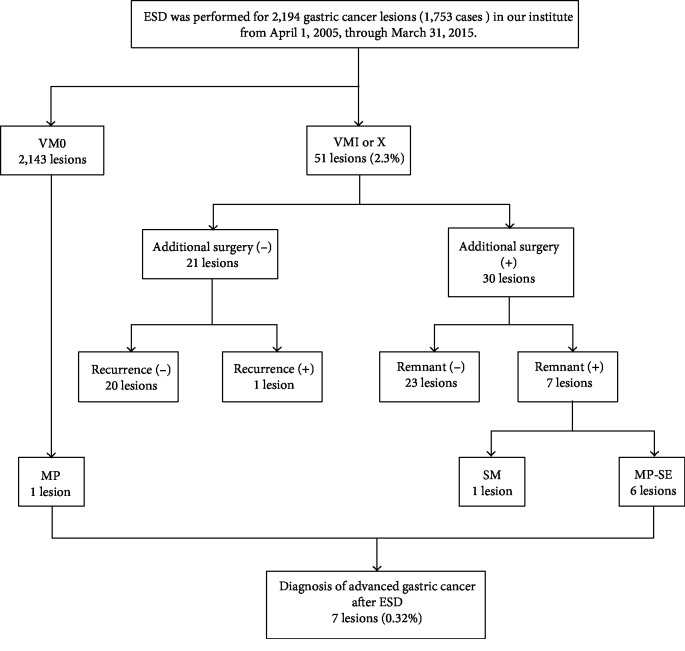
Flow chart of lesion selection. ESD: endoscopic submucosal dissection; MP: muscularis propria; SE: serosa exposed; SM: submucosa; VM: vertical margin.

This was a single-center retrospective study approved by the ethics committee of Toranomon Hospital and performed based on the ethical principles of the Declaration of Helsinki for medical research involving human subjects. ESD was performed for 2,194 gastric cancer lesions (1,753 cases) in our institute from April 1, 2005, through March 31, 2015. Of these, 2,143 lesions (97.7%) were resected with negative margins. The vertical margin was positive or status unknown in 50 cases (51 lesions, 2.3%). Additional surgery was performed on 29 of these cases (30 lesions). Pathological findings after these additional surgeries showed tumor remnant in 7 cases (7 lesions). The remnant tumor was in the SM layer in 1 case, and MP invasion was found in 6 cases (6 lesions). MP invasion was also found in 1 lesion out of the 2,143 in which the vertical margin was negative. Clinicopathological examination was performed on a total of 7 lesions for which the final pathological results showed invasion beyond the MP layer (Figure 1).

Endoscopic examination was performed every 6 months to 1 year after surgery. Computed tomography examination and abdominal ultrasonography were also performed at this interval to evaluate for distant metastasis.

### 2.2. Endoscopic Submucosal Dissection Procedure

For 5 of 7 lesions, patients underwent ESD under sedation with phthalidine hydrochloride and diazepam. For the remaining 2 lesions, ESD was performed under general anesthesia.

ESD was generally performed using a flex knife or dual knife (Olympus Optical Co., Ltd., Tokyo, Japan), with a hook knife (Olympus, Japan) used at the discretion of the operator. The high-frequency device used for ESD was an ICC 200 or VIO 300 D (ERBE GmbH, Tübingen, Germany), and the endoscope was generally a GIF-2TQ260M (Olympus, Japan), with a GIF-Q260J used in combination at the discretion of the operator. Marks were placed around the lesion, and an additional mark was placed on one side to indicate the direction of the lesion. A mucosal incision was made after injection of fructose-added glycerol solution (Glycerol; Chugai Pharmaceutical Co., Tokyo, Japan) or hyaluronic acid. An additional injection was administered into the submucosal layer if necessary, after which, dissection was performed. Finally, an *en bloc* resection was performed.

### 2.3. Endoscopic Analysis

A GIF-Q240Z or GIF-H260Z endoscope (Olympus, Japan) was used to perform endoscopic diagnosis of lesions by an expert with experience in over 1,000 endoscopic diagnoses for early gastric cancer using EUS and NBI-ME. First, endoscopic diagnosis before ESD and pathological diagnosis of ESD specimens and surgical specimens were reviewed retrospectively. Second, endoscopic images were reviewed retrospectively by 3 endoscopists in white light, NBI-ME, and EUS to evaluate for endoscopic findings of SM invasion. Two of the 3 endoscopists were members of the Japanese Gastroenterological Endoscopy Society; the other was a trainee. When opinions differed, the final endoscopic diagnosis was determined by majority decision.

Four findings from white light images were used to calculate the depth-predicting score [5]: margin elevation (2 points), tumor size greater than 30 mm (2 points), remarkable redness (1 point), and uneven surface (1 point). The presence or absence of a dilated blood vessel (D vessel) on the tumor surface was evaluated in NBI-ME [6]. Obvious irregular narrowing or budding into the third sonographic layer [7, 8] was evaluated in EUS as an index of SM invasion. All EUS scans were performed at 20 MHz.

### 2.4. Pathological Analysis

ESD specimens were sliced at 2 mm intervals and subjected to hematoxylin and eosin (HE) staining. Surgical specimens were sliced at 5 mm intervals and subjected to HE staining. Immunostaining was performed if necessary. Pathological evaluation was based on the Japanese Classification of Gastric Carcinoma [9, 10]. Findings from pathological diagnosis of the ESD specimen and surgical specimen were combined to obtain the final pathological diagnosis. In addition, the lesion was mapped onto both the ESD specimen and the surgical specimen, and the area of cancer cells exposed on the mucous surface (mucosal exposed area) and the area in which cancer cells were found in either the mucosal layer or deeper than the submucosal layer (total lesion area) were measured using ImageJ image processing software (National Institutes of Health, Bethesda, MD). The ratio of total lesion area to mucosal exposed area was then calculated.

## 3. Results

### 3.1. Patient Characteristics and Endoscopic Diagnosis before Endoscopic Submucosal Dissection

The 7 lesions diagnosed as advanced cancer represented 0.32% of all gastric cancer lesions for which ESD was performed in our hospital from April 1, 2005, through March 31, 2015. The average age of the 7 cases was 73.2 ± 7.2 years; all were male. All lesions were found in the U region (fundus). There were 5 cases with no prior treatment, 1 case of recurrence after endoscopic mucosal resection (EMR), and 1 case after esophagectomy with gastric pull-up. Pre-ESD biopsy results included 1 undifferentiated type, 1 differentiated dominant type, 4 differentiated types, and 1 group 4. Macroscopic types based on endoscopic diagnosis before ESD were 0-IIa in 2 cases, 0-IIc in 4 cases, and 0-III in 1 case. Depth prediction before ESD was M for 6 cases and SM1 for 1 case. The estimated diameter of the lesions was 32.9 ± 27.6 mm on average. Ulcers were found on endoscopy in 3 of 7 cases (Table 1).

### 3.2. Retrospective Review of Endoscopic Images

**Figure 2:**
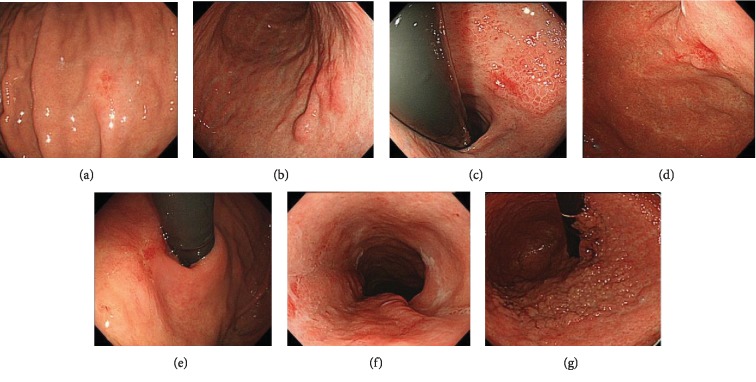
White light images of all lesions: (a) lesion ① in Tables 1–4; (b) lesion ② in Tables 1–4; (c) lesion ③ in Tables 1–4; (d) lesion ④ in Tables 1–4; (e) lesion ⑤ in Tables 1–4; (f) lesion ⑥ in Tables 1–4; (g) lesion ⑦ in Tables 1–4.

Scoring for white light endoscopic findings (margin elevation, tumor size, uneven surface, and remarkable redness) was 0 points in 2 cases, 2 points in 3 cases, and 3 points in 2 cases; therefore, 2 of 7 cases had a score of 3 or more, indicating deeper SM cancer (Figure 2).

**Figure 3:**
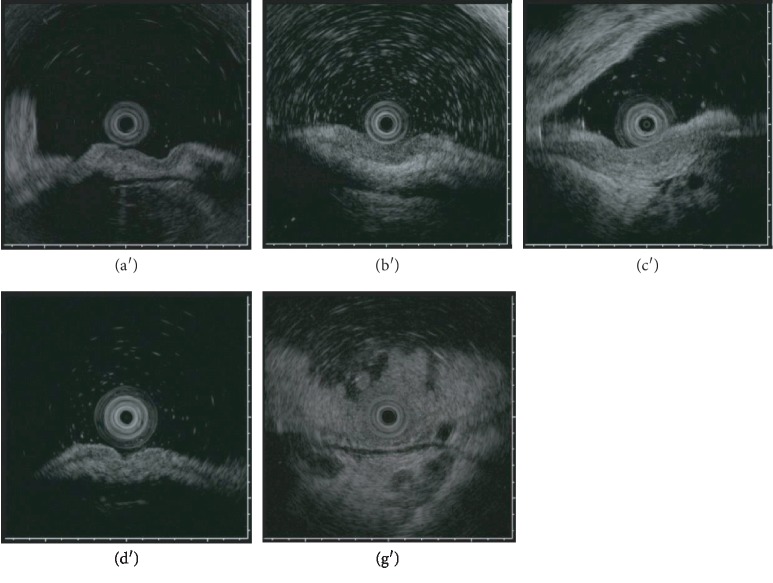
Endoscopic ultrasonography images of 5 lesions: (a′) lesion ① in Tables 1–4; (b′) lesion ② in Tables 1–4; (c′) lesion ③ in Tables 1–4; (d′) lesion ④ in Tables 1–4; (g′) lesion ⑦ in Tables 1–4.

NBI-ME was performed in 6 cases; of these, D vessels were found in 3 cases. Of the 5 cases in which EUS was performed, obvious irregular narrowing or budding into the third sonographic layer was observed in 2 cases (Figure 3).

No cases showed evidence of deeper SM cancer in all 3 modalities (white light endoscopy, NBI-ME, and EUS) upon retrospective review (Table 2).

### 3.3. Pathological Analysis

Table 3 shows the pathological diagnoses of the ESD specimens and surgical specimens and the combined final pathological diagnoses.

**Figure 4:**
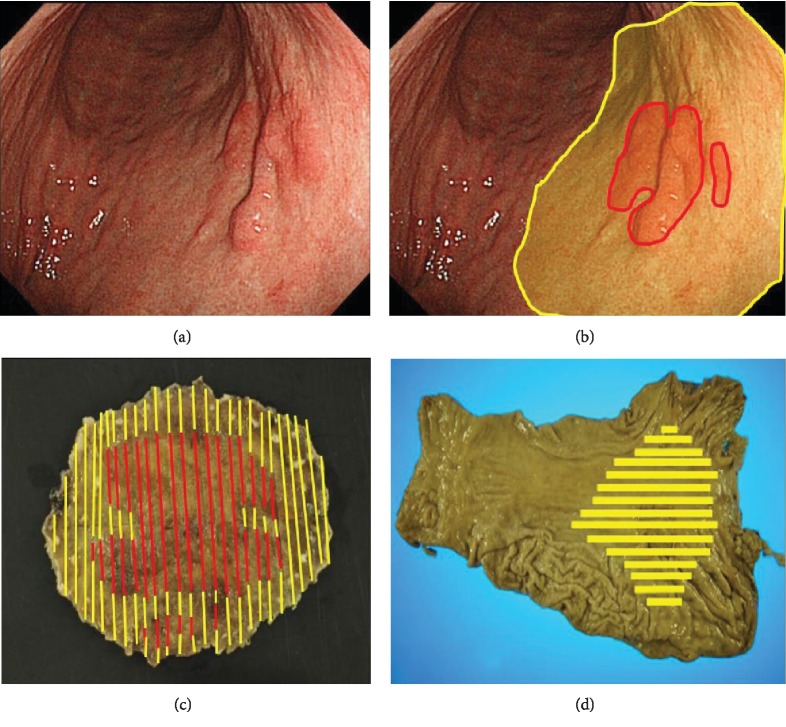
One case of advanced gastric cancer (pT4a) in which ESD was performed. (Lesion ② in Tables 1–4). (a) White light image. (b) Combined image of cancers by ESD and surgical pathology. (c) Cancer area mapped onto ESD specimen. (d) Cancer area mapped onto surgical specimen. (Red area shows cancer area exposed on the mucous surface and yellow area shows cancer area spread in the SM and MP layers in images (b)–(d)).

In the final pathological results, the average tumor diameter was 60 ± 49 mm and there was 1 case of differentiated cancer, 2 cases of mixed differentiated dominant cancer, 2 cases of mixed undifferentiated dominant cancer, and 2 cases of undifferentiated cancer. Of the 7 cases, tumor depth was T2 for 4 cases, T3 for 2 cases, and T4a for 1 case (Figure 4).

The type of invasion was INFc in all cases. Lymphovascular invasion and lymph node metastasis were observed in 6 cases and 3 cases, respectively. The total lesion area was larger than the mucosal exposed area in 6 of 7 cases (i.e., most cases had an area in which the cancer was not exposed to the mucosal layer but had spread in the SM and MP layers). In 1 case, partial MP infiltration was observed in part of a large, flat, raised lesion. The average ratio of total lesion area to mucosal exposed area was 3.18 ± 2.47 (Table 4).

### 3.4. Patient Prognosis

The average observation period was 1,411 ± 1,084 days. During that time, no recurrence or death from primary disease was observed among the evaluated cases.

## 4. Discussion

ESD is widely used as a minimally invasive treatment for early gastric cancer. The merits of ESD include *en bloc* resection, low risk of local recurrence, and precise pathological evaluation. Large lesions that are difficult to remove completely by conventional EMR and lesions with ulcer scars can be removed *en bloc* using ESD. While indications for ESD have increased in recent years, the procedure is targeted for early-stage cancer with intramucosal or submucosal invasion depth. Current guidelines include curative resection of predominantly differentiated tumors up to 3 cm in diameter and of invasion depths up to SM1 (extending less than 500 *μ*m into the SM layer) as an expanded indication [2]. For this reason, endoscopic studies have been conducted to identify differences between lesions that are intramucosal or infiltrate the submucosal superficial layer and those that infiltrate the deeper submucosal region [5]. However, the present study revealed several cases in which lesions resected using ESD after a diagnosis of early-stage cancer were found to have infiltrated beyond the MP layer, although this was rare (in our hospital, approximately 0.32% of cases over 10 years).

Endoscopic depth diagnosis of early gastric cancer has conventionally been performed using white light endoscopy and EUS, but the accuracy of this approach is limited. Observation of gross morphology with white light, EUS, and NBI-ME during depth diagnosis is important for accurate determination of whether a particular lesion is actually indicated for ESD.

Five lesions out of 7 cases in this study were depressed type, consistent with reports that the flat or depressed form of cancer is an independent factor affecting the accuracy of depth diagnosis [11]. In addition, ulceration (UL) findings were noted in white light endoscopy in 1 of 7 cases. Endoscopic findings of lesions with UL—including submucosal tumor-like marginal elevation due to inflammation or edema, and fibrosis due to ulcer scarring—have been described as similar to findings of lesions with SM invasion. Therefore, it is considered difficult to accurately determine invasion depth in lesions with UL.

Namieno et al. reported that depth diagnosis of surgically resected specimens was significantly less accurate in patients with versus without endoscopic UL [12]. In the present study, the 1 case with UL was tub1 > tub2 in histology, but its depth was T3; thus, UL findings should be carefully noted. On the other hand, there were 2 cases with 0-IIa lesions. Most 0-IIa lesions are considered to be M cancers, and advanced cancers are rare. Fujisaki et al. reported that 85% of surgically resected 0-IIa lesions under 20 mm in diameter were M cancer; however, half of those over 51 mm in diameter were SM. Furthermore, 67% of 0-IIa lesions over 41 mm in diameter in ESD cases were SM cancer [13]. The 0-IIa lesions in the present study were as large as 50 mm and 80 mm, respectively. In addition, scoring of 4 white light endoscopy findings related to SM2 cancer [5] revealed 5 cases (71.2%) with scores of 0–2 points, which generally can be diagnosed as M-SM1 cancer with high accuracy. These findings suggest that most cases in this study would be difficult to diagnose as SM2 or more advanced cancer by white light endoscopy.

Regarding ultrasound endoscopy, Watari et al. [14] reported that there was no significant difference in diagnosis accuracy between differentiated and undifferentiated types when predicting the invasion depth of early gastric cancer by EUS. On the basis of their report, we speculate that the lesions in the present study are not typical and may include linitis plastica (LP), a poorly differentiated cancer that diffusely invades the gastric wall and causes fibrosis [15]. Of the 4 cases that had no UL findings in EUS, 3 were considered to be similar to LP-type gastric cancer and 1 was found to be a difficult-to-diagnose LP-type gastric cancer [16]. Moreover, while some fibrosis in LP-type gastric cancer can be recognized as low-echoic lesions in EUS, deeper invading scirrhous cancer cells may be difficult to recognize [17]. In cases without UL, 1 case pathologically categorized as por2 > sig was difficult to evaluate in EUS because of individual cancer cell invasion, and 1 case categorized as tub2 > por2 was 0-IIa type with a lesion so large (80 mm in diameter) that it would be difficult to evaluate the entire lesion on EUS.

The presence of D vessels was observed in 3 out of 6 cases that underwent NBI-ME; however, there is no consensus on the usefulness of magnifying endoscopy for depth diagnosis of gastric cancer [18].

As mentioned above, no lesions had findings associated with SM cancer in all 3 modalities (white light, EUS, and NBI). The lesions could be divided into 3 groups based on endoscopic findings: (1) strongly reddish 0-IIc lesions or 0-III lesions, (2) large 0-IIa lesions, and (3) lesions with previous treatment history before ESD or 0-IIc lesions on the suture line. The 7 cases in this study can also be roughly divided into 3 types pathologically: (a) undifferentiated type (so-called LP type) in which the infiltrated area in the submucosa was larger than the exposed area on the mucosal surface, (b) lesions with a pretreatment scar or ulcer scar, and (c) special cases. In group (a), the pathological results of pre-ESD biopsies were the undifferentiated type, undifferentiated mixed type, or group 4. Moreover, in the final pathological results, the infiltrated area in the MP layer or deeper was por2 > sig and the infiltrating type was INFc. Cases in group (b) may be further divided into 3 subtypes: lesions recurring after EMR, lesions with peptic ulcers, and lesions on the suture line of the gastric tube. In this group, all pre-ESD biopsy findings indicated a differentiated type, while the final pathological results showed that 2 lesions were a differentiated type and 1 was por2 in the infiltrating area. Group (c) comprised a special case in which a wide (142 mm × 100 mm) papillary adenocarcinoma was resected by ESD. In this specimen, the muscle layer was partly resected and there was a small infiltrated area of tub2 > por2.

Interestingly, all 7 cases were located in the U region of the stomach. This might be because the tumor depth of lesions in the U region is difficult to diagnose correctly by endoscopy. Furthermore, 4 lesions involved the esophagogastric junction, and cancer in this area has been reported to have significantly more submucosal invasion, lymphatic vessels, and venous invasion [19]. Together, these factors could help explain why the initial biopsy findings for the 7 lesions in this study differed substantially from the final diagnosis.

There are no previous reports of cases in which MP invasion or deeper was found in lesions for which ESD was thought to be indicated. To our knowledge, the present study is the first to clarify the proportion of lesions for which endoscopic depth diagnosis and pathological depth diagnosis differ substantially and to examine the clinicopathological features of these lesions. The lesions identified in this study are thought to be special cases in which it was difficult to identify tumor infiltration deeper than MP, even with the use of multiple endoscopy modalities. Although such cases are rare, other facilities are likely to encounter a number of these lesions over time.

The chief limitations of this study are its single-center retrospective design and small number of cases. Furthermore, it is unclear whether patients for whom additional surgery was not performed despite vertical margin- (VM-) positive findings in ESD pathology could be target cases. For these patients, it is difficult to determine whether cancer cells were already present in the muscle layer at the time of ESD or whether the muscle layer was infiltrated after a recurrence. In fact, 1 of the 21 cases in this study who were VM-positive but did not undergo additional surgery had a local recurrence; however, it could not be determined whether this patient had advanced cancer, as he was elderly and therefore did not receive surgical treatment after the recurrence. It may be possible to elucidate additional details by accumulating and analyzing similar cases.

## 5. Conclusions

We identified 7 cases, 0.32% of the total, in which gastric cancer lesions were found to be deeper than T2 in the final pathological diagnosis despite being previously determined to be indicated for ESD. Each case in this study had a unique set of characteristics that prevented accurate diagnosis using various endoscopic modalities.

## Figures and Tables

**Table 1:** Clinical diagnosis of 7 lesions before endoscopic submucosal dissection.

Lesion	Tumor diameter	Depth	Gross morphology	UL	Histology of biopsy
①	10 mm	M	0-IIc	−	por2 > sig
②	50 mm	SM1	0-IIa	−	tub2 > por
③	10 mm	M	0-IIc	−	Group 4
④	10 mm	M	0-III	+ (peptic ulcer)	tub1 > tub2
⑤	10 mm	M	0-IIc	+ (EMR scar)	tub2
⑥	60 mm	M	0-IIc	+ (suture line)	tub1 > tub2
⑦	80 mm	M	0-IIa	−	pap

EMR: endoscopic mucosal resection; M: mucosal cancer; SM: submucosal cancer; UL: ulcer.

**Table 2:** Retrospective review of endoscopic findings.

Lesion	White light image						EUS	NBI-ME
	Remarkable redness	Uneven surface (nodulation)	Margin elevation	Size > 30 mm	Total score	Depth-predicting score ≥ 3	Obvious irregular narrowing or budding into third sonographic layer	D vessel
①	−	−	−	−	0	×	+	−
②	+	−	−	+	3	〇	−	+
③	−	−	+	−	2	×	−	+
④	+	−	+	−	3	〇	−	−
⑤	−	−	−	−	0	×	N/A	N/A
⑥	−	−	−	+	2	×	N/A	+
⑦	−	−	−	+	2	×	+	−

EUS: endoscopic ultrasonography; NBI-ME: narrow-band imaging magnifying endoscopy; N/A: not available.

**Table 3:** Final pathological diagnosis after additional surgery.

Lesion	Location	Tumor size	Histological type (mucosal exposed area)	Histological type (infiltration area)	Depth of tumor invasion	Cancer stromal volume	Infiltrative pattern	Lymphovascular invasion	Lymph node metastasis
①	U, post	50 × 35 mm	por2 > sig	por2 > sig	T2	sci	c	ly0, v0	N1
②	UM, post	120 × 115 mm	tub2 > por	por2 > sig	T4a	sci	c	ly1, v1	N2
③	U, ant	35 × 22 mm	tub2 > por2 > sig	por2 > sig	T2	sci	c	ly1, v0	N0
④	U, post	25 × 20 mm	tub1 > tub2	tub1 > tub2	T3	int	c	ly0, v1	N0
⑤	U, less	30 × 15 mm	tub1	tub1	T2	int	c	ly1, v0	N0
⑥	U, post (gastric tube)	86 × 33 mm	tub2	por2	T3	sci	c	ly2, v1	N1
⑦	UE, less	142 × 100 mm	pap > tub2	tub2 > por2	T2	sci	c	ly2, v1	N0

Ant: anterior wall; E: esophagus; int: infiltration; less: lesser curvature; M: corpus; post: posterior wall; sci: scirrhous type; U: fundus.

**Table 4:** Areas and ratio of total lesion area to exposed mucosal area.

Lesion	Mucosal exposed area (cm^2^)	Total lesion area (cm^2^)	Ratio of total lesion area to mucosal exposed area
①	4.03	11.83	2.94
②	12.53	111.11	8.87
③	3.02	4.63	1.54
④	1.68	2.68	1.6
⑤	0.76	2.13	2.8
⑥	5.85	20.58	3.51
⑦	110.09	110.09	1
Average	19.71	37.58	3.18

## Data Availability

The data used to support the findings of this study are available from the corresponding author upon reasonable request.
